# The greatest contribution to medical science is the transformation from studying symptoms to studying their causes—the unrelenting legacy of Robert Koch and Louis Pasteur—and a causality perspective to approach a definition of SLE

**DOI:** 10.3389/fimmu.2024.1346619

**Published:** 2024-02-01

**Authors:** Ole Petter Rekvig

**Affiliations:** ^1^ Section for Autoimmunity, Fürst Medical Laboratory, Oslo, Norway; ^2^ Department of Medical Biology, Faculty of Health Sciences, UiT The Arctic University of Norway, Tromsø, Norway

**Keywords:** systemic lupus erythematosus, classification criteria, tentative diagnostic criteria, anti-dsDNA antibodies, lupus nephritis, forensic science definitions, hard evidence, circumstantial evidence

## Abstract

The basic initiative related to this study is derived from the fact that systemic lupus erythematosus (SLE) is a unique and fertile *system science* subject. We are, however, still far from understanding its nature. It may be fair to indicate that we are spending more time and resources on studying the complexity of *classified* SLE than studying the validity of classification criteria. This study represents a theoretical analysis of current instinctual^1^ SLE classification criteria based on “the causality principle.” The discussion has its basis on the radical scientific traditions introduced by Robert Koch and Louis Pasteur. They announced significant changes in our thinking of disease etiology through the implementation of the modern version of “the causality principle.” They influenced all aspects of today’s medical concepts and research: *the transformation of medical science from studies of symptoms to study their causes*, relevant for monosymptomatic diseases as for syndromes. Their studies focused on bacteria as causes of infectious diseases and on how the immune system adapts to control and prevent contagious spreading. This is the most significant paradigm shift in the modern history of medicine and resulted in radical changes in our view of the immune system. They described *acquired post-infection immunity* and *active immunization by antigen-specific vaccines*. The paradigm “transformation” has a great theoretical impact also on current studies of autoimmune diseases like SLE: *symptoms and their cause(s)*. In this study, the evolution of SLE classification and diagnostic criteria is discussed from “the causality principle” perspective, and if contemporary SLE classification criteria are as useful as believed today for SLE research. This skepticism is based on the fact that *classification criteria are not selected based on cogent causal strategies*. The SLE classification criteria do not harmonize with Koch’s and Pasteur’s causality principle paradigms and not with Witebsky’s Koch-derived postulates for autoimmune and infectious diseases. It is not established whether the classification criteria can separate SLE as a “one disease entity” from “SLE-like non-SLE disorders”—the latter in terms of SLE imitations. This is discussed here in terms of weight, rank, and impact of the classification criteria: Do they all originate from “one basic causal etiology”? Probably not.

## 1 Introduction

If we are going to solve problems in systemic lupus erythematosus (SLE) research, we have to as first priority identify, describe, and argue for their nature, causality, and legacy (1–8). Only then can we solve them and bring insight and consequences further. This is a valid perspective relevant to system science in general and to SLE in particular. Still, we describe SLE as an enigmatic autoimmune syndrome. After all these years, we have to define the basis for its position as an enigmatic syndrome. Although the elements of the syndrome have been solved or are close to be described—like the origin and diversity of anti-dsDNA antibodies and the genesis of lupus nephritis and cerebral lupus—the basis for the composition of the elements (here: criteria) remains unsolved. For example, criteria count regardless of *when* they timely occur. This is clearly in conflict with Koch’s causality criteria for an infectious disease or from Witebsky’s derivative of Koch’s criteria applied to autoimmune diseases (see below). This approach is attempted in this study. The core question is whether we have developed algorithms that will allow us to identify the basis for central problems. Moreover, the central questions are: Are the current criteria useful as algorithms that may solve pathobiological problems in SLE? Are we sufficiently open-minded to develop radical solvable hypotheses? Can we circumvent or neglect the causality principle in this context?

SLE is defined by classification criteria. These criteria are elaborated through several steps: First, a large panel of criteria thought to define pathogenic processes that characterize SLE were *selected.* The selection process was fulfilled by an expert panel and was in principle intuitively based on traditions, experience, and insight, but was not discussed in a causal context [see the original reports (9–12)]. Among these criteria, a final set of operational SLE classification criteria was *elected* by Delphi panels. Finally, the elected classification criteria panel was statistically *legitimated* by comparing them with former classification criteria sets (9–12). The *validity* of this process is not critically discussed in the relevant literature but will be analyzed in this study.

The processes leading to SLE classification criteria are largely based on principles today as those described 50 years ago. These were described in “The preliminary SLE classification criteria” in 1971 and in all subsequent classification criteria versions [see (9–12)]. The problematic part is that the attempts to define the nature and the inner coherence of central molecular and/or genetic etiologies responsible for early and progressive SLE are not implemented in those canonical processes: The criteria selection process is not anchored in the causality principle.

A missing link in our understanding is if the classification criteria (as effects) are instigated by a dominant cause or by a series of causes linked in a downstream pattern and basically instigated by a main fundamentally causal origin or if individual criteria are effects of unique individual causes unlinked from each other. In the latter example, it is difficult to understand how they contribute to the “genuine” syndrome SLE and not to “SLE-like non-SLE” syndromes.

In his masterful narrative, Paul de Kruif describes, engaged and devoted in his book “The Microbe Hunters,” a new and radical paradigm shift in the history of medicine (13). He illustrates how Robert Koch and Louis Pasteur simultaneously—and competitively (the epic man against man, Germany against France)—described bacteria as the principal cause of infectious diseases (13). They and their successors, particularly, Emile Roux, Emil Behring, and Paul Ehrlich, uncovered the principles of acquired (post-infection) and induced (vaccination) immunity against infectious agents. A consequence of these achievements is that their radical paradigms to control and fight infectious diseases and to limit epidemics developed into a new and still central doctrine: *Transformation of medical science from studying symptoms to studying their causes—and how to protect against these causes* (13). This was simply the birth of the modern version of “*the causality principle*” in medical science. “The causality principle” is thought-provokingly discussed by Clarke et al. (14) and is remarkably concrete and philosophically contemplated by Bunge in his text “Causality and modern science” (15). This imperative scientific principle has, today more than ever, a significant impact on how to study cause–symptom and cause–criterion relationships in diseases. This is also highly relevant for enigmatic autoimmune syndromes.

## 2 The legacy of systemic lupus erythematosus and its *status praesens*

SLE is one of these enigmatic autoimmune syndromes. SLE is still provisionally classified as an “enigmatic (and a prototype)” autoimmune syndrome [see, e.g (1, 4, 16–19)]. Due to its enigmatic character, current research activities aim to describe SLE by analytical methods that cross many scientific borders—like genetics [monogenic (20–23) and polygenic (24–26) expression profiles], humoral and cellular immunity versus tolerance regulation, impact of autoimmunity, microbiota, pathophysiology, inflammation and inflammatory mediators, gender, clinical medicine, and statistical methods. In this respect, SLE has for scientists been a fertile, challenging, and learning topic. From these scientific activities, considerable achievements and consequent insight into molecular and cellular biology, and into the regulation and function of the immune system, have contributed to the enormous inspiration and interest in SLE worldwide [see, e.g (27).,]. Much of these conglomerated research activities originate from *in-vitro* analyses and from studies of SLE patients enrolled into cohorts by authorities of SLE classification criteria (9–12, 28–31). This latter statement represents scientific challenges as these criteria are not exact measures that concisely describe SLE as a definable existent syndrome. This is discussed in detail below.

SLE classification criteria are claimed to define SLE as a “disease entity” (31). It is, however, problematic to use the “entity” terminology in this context without defining what is meant semantically, contextually, and in the end how to describe SLE as a holistic and integrated syndrome [discussed in (4, 32)]. The perception of SLE as a disease entity is somewhat immature related to our incomplete understanding of its etiology and is today critically challenged by current interdisciplinary research directions [see an overview in (27), exemplified in (33)]. Altogether, these scientific elements represent the interference of different contrasting scientific fields and their systematic and analytical implementations. Considering this complex pathophysiological picture and the *de-facto* lack of implementation of the causality principle in the generation of SLE classification criteria (see below), we are still far from formally accepting SLE as a disease entity. It may hence be relevant to conclude that contemporary SLE cohorts are not logically composed and described and probably not sufficiently homogeneous to serve as substrates for studies of the syndrome’s basic etiology and cause-reflecting pathophysiology [see also critical comments by Tsokos (3) and a detailed discussion below]. SLE cohorts as those established today may be composed of patients suffering from “genuine SLE,” “the one etiology-disparate phenotypes-one disease entity” paradigm, and “SLE-like non-SLE” versions of the syndrome (see below).

**Figure 1:**
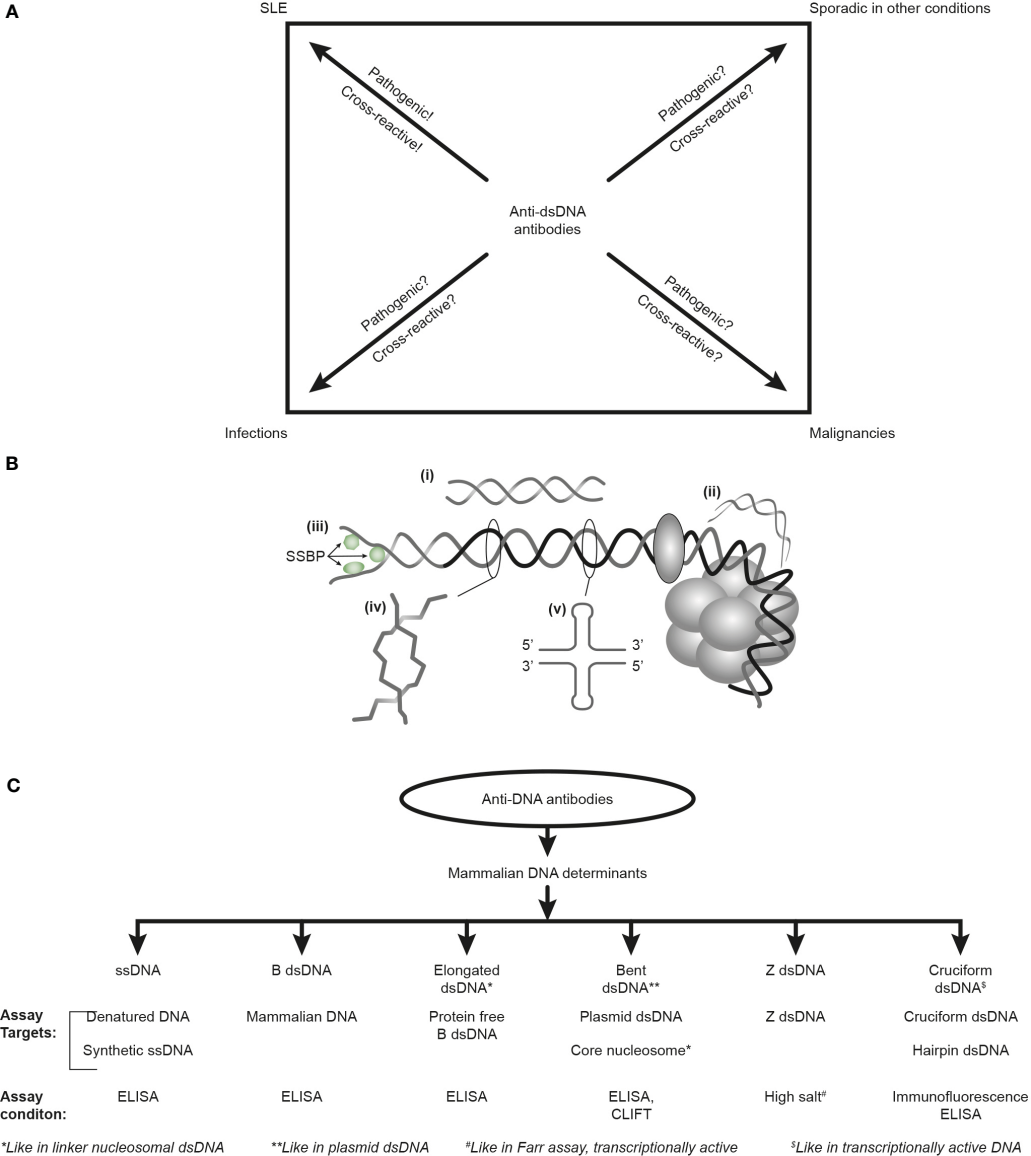
Anti-DNA antibodies: diagnostic impact, specificity for unique DNA structures, and assays. The anti-dsDNA antibodies are not unique for systemic lupus erythematosus (SLE) but appear regularly in the context of infections and malignancies and sporadically in other conditions **(A)**. Whether anti-dsDNA antibodies are pathogenic and cross-reactive in the latter conditions is not thoroughly investigated [question marks in **(A)**], as they truly are in SLE. Critical questions can be raised against the SLE classification criterion “the anti-dsDNA antibody” (criterion 11 in ACR) or “anti-dsDNA” (criterion 6, immunological criteria, SLICC). This terminology indicates that the anti-dsDNA antibody is one (polyclonal) antibody with one specificity without further detailed information. This has over decades precipitated the concept that different assay systems detect antibodies possessing different avidities but not different specificities! The correct information is that DNA in chromatin expresses distinct DNA structures, each having distinct functions, and each structure is a unique antigenic determinant **(B)**. Elongated (linker) DNA is a relaxed, right-handed low-energy linear form of B-DNA **(i)**, while compacted B-DNA as in plasmids (not shown) and in core nucleosomes are defined as bent B-DNA (ii). In (iii), the B-DNA helix is opened by single-stranded DNA-binding proteins (SSBP, i.e., proteins stabilizing ssDNA and promoting access for polymerases involved in replication and repair). In (iv), Z-DNA is demonstrated, which is a left-handed, high-energy, supercoiled double helix. Z-DNA is predominantly associated with linker DNA and regulates transcription. Cruciform DNA is another structure formed in dsDNA (v) and is different from B- and Z-DNA. The cruciform structures are, like Z-DNA, higher-energy DNA structures. From an immunogenic point of view, each structure (i–v) is unique in terms of inducing highly specific antibodies with potential pathogenic impact if chromatin is exposed *in situ*. Antibodies against all these dsDNA structures have been identified by conventional but discriminating assay systems **(C)**. Data presented in this figure require that assay systems for anti-dsDNA antibodies relate to categorized structural DNA specificities. The lack of implementation of the structural DNA recognition pattern of individual anti-dsDNA antibodies may undermine attempts to define the potential clinical (diagnostic and pathogenic) impact of anti-dsDNA antibody subspecificities. This figure is modified and reorganized and is a fusion of the instructive **Figure 1** and **2** in reference (34).

Furthermore, the factual unsophisticated concept of SLE as a syndrome *diagnosed* by, e.g., anti-dsDNA antibodies, is incomprehensible as these antibodies appear frequently in conditions like infections, malignancies, and other diseases [see **Figure 1A** (6, 34, 35) but consider an opposite or alternative view in (8)]. Anti-dsDNA antibodies may therefore alternatively be regarded as a subordinated diagnostic factor. This is discussed below in the context of their *pathogenic rather than their diagnostic impact* in SLE.

If we read the original publications (10–12) [autoimmunity was not implemented in the first set of preliminary SLE classification criteria (9)] and interpret them contextually, this will clearly lead to a discussion on whether or not anti-dsDNA autoimmunity is a central element linked to the basic causality principle in SLE. Anti-dsDNA antibodies have, nevertheless, an important function as a *disease-modifying factor* in SLE. These considerations affect our strategy aimed at defining SLE.

## 3 Clinical impact of anti-dsDNA autoantibodies in SLE—are they all always diagnostic and pathogenic?

We have an immediate and imperative proclamation: *Anti-dsDNA antibodies are not always pathogenic in SLE and they are not always diagnostic for SLE.* They are pathogenic only when chromatin fragments are exposed extracellularly in, e.g., basement membranes, or alternatively, when they cross-react with intrinsic non-DNA membrane structures (see below).

Exposure of chromatin fragments in glomeruli is enhanced by silencing of the renal DNase 1 gene and a consequently reduced fragmentation of chromatin from dead cells (36–38). This has been shown to create anti-dsDNA antibody–chromatin fragment immune complex formation and immune complex-mediated glomerular tissue inflammation (36). Notably, such complexes may be targeted by heparin treatment [see below (5, 39)]. Therefore, the combination of increased titers of serum anti-dsDNA antibodies (6, 35) and reduced renal DNase 1 enzyme activity (36, 37), as is reflected by reduced renal DNase 1 levels also in urine samples (40), represents a complex causal element in progressive lupus nephritis. Increased anti-dsDNA antibody activities and loss of renal DNase 1 endonucleolytic function are therefore, if they appear combined, a clear complex candidate *classification and diagnostic criterion* for lupus nephritis and for SLE itself. It is in this respect important to state that both detection of anti-dsDNA antibodies and a combined quantification of anti-dsDNA antibodies and urinary DNase 1 levels are easy to perform (40) and, therefore, unproblematic to implement in clinical routine laboratories. *This is a direct implementation of the causality principle in SLE —its logic—and explains the pathogenesis of lupus nephritis.* The pure presence of anti-dsDNA antibodies in abnormal titers, as stated in the classification criteria, is a rather modest single classification criterion. These antibodies may by themselves not always predict the active pathophysiological processes characteristic of SLE [ (6, 34, 35), discussed below] and are not even convincingly diagnostic for SLE (6).

Whether contemporary SLE cohorts are useful as objectives for investigating SLE etiology and pathogenesis is a case for principal discussions. The basic argument for this critical proposition is that we are still not able to present a solid and cause-related definition of SLE [see discussions in (1–4, 16)].

Today, published studies often introduce SLE as a “prototype systemic autoimmune syndrome.” Is this dogma true? Is it scientifically sound to assume that SLE classification criteria as those we know today are of fundamental value to establish SLE cohorts for further investigations of this systemic autoimmune syndrome (3, 45)? Maybe we should consider SLE defined by current classification criteria as a syndrome that represents a group of disorders that is influenced also by imitations (46, 47). These latter syndromes may be denoted “SLE-like non-SLE syndromes” to demarcate them from a “genuine causal-driven SLE.” If so, “SLE-like non-SLE syndromes” represent manifestations of diffuse etiologies and are not connected to a cause-related inflammatory network that in this context classifies “genuine” SLE. *“Genuine” indicates here SLE as a potentially explainable syndrome defined by its cause(s)*. In other words, we see in the present context the contour of a conflict between *phenomenology* and *causality* that makes SLE cohorts heterogeneous for penetrating studies of SLE.

Heterogeneity is a term that may not be helpful in the context of the present study. SLE is heterogenic—but is it so because the basic disease process (the cause) promotes a polyphenotypic (heterogenic) syndrome or is it so because the classification criteria are non-stringent and principally not elaborated in the context of the causality principle? This eventually involves criteria that may be regarded as second-order criteria (see below) that may lead to SLE diagnoses like “SLE-like non-SLE” syndromes. For example, SLE cohorts incorporate patients with and without anti-dsDNA antibodies, lupus nephritis, cerebral lupus, alopecia, arthritis, and so on. This can theoretically lead to high numbers of SLE phenotypes. For example, the 1982 ACR SLE classification criteria collection consists of 11 items. Of these, four must be recorded to enroll a patient in an SLE cohort; this may at maximum lead to 330 different SLE phenotypes. Do all these phenotypes constitute “genuine” SLE? This is further discussed in detail below.

## 4 Underestimating basic theories for causality and objectivity harms scientific practice: a need to introduce the causality principle perspective to reveal concrete and coherent SLE criteria

The first part of this heading has been a leading star in this author’s scientific life and has made science complicated! Causality (15, 48) and objectivity (49, 50) are elements that ideally direct all scientific elements from the generation of hypotheses, via conducting controlled experiments, to interpretations and objectively probing the results logically and statistically.

From what has been the main focus in recent manuscripts (4–6, 34, 40, 45) is an appreciation of the simple fact that we have *not* comprehended or invented a firm definition of SLE and its basic etiology. This reminds on earlier—today’s obsolete—dogmas that stated that i) mammalian dsDNA was basically non-immunogenic (51–54); ii) lupus nephritis was caused by cross-reactive anti-dsDNA antibodies recognizing intrinsic membrane constituents like laminin, entactin, collagen, or other non-DNA ligands [**Table 1** (55–78), see below]; iii) anti-dsDNA antibodies in clinical medicine expressed one molecular DNA specificity (“The anti-dsDNA antibody”) included in the classification criteria without any further specification or dissection of the term [**Table 1** (10–12), critically discussed in (34), see also below]; and iv) anti-dsDNA antibodies were highly diagnostic for SLE although published data tell a quite different story [see, e.g (6, 35, 43, 44, 79–84), discussed in (33)].

**Table 1:** Examples of anti-dsDNA antibodies that cross-react with non-DNA structures.

Anti-dsDNA antibody cross-react with	References
α-Actinin	(55)
α-Actinin Laminin	(56) (57)
C1q	(58)
Several cross-reactive activities presented at the “Fifth International Workshop on anti-DNA antibodies in London 2002 to highlight relevant properties of pathogenic anti-DNA antibodies”	(59)
Laminin	(60)
Nucleosomes	(61)
Platelet integrin GPIIIa 49-66	(62)
TLR 4	(63)
NR2 glutamate receptor	(64)
Cell surface proteins	(65)
Ribosomal P protein	(66)
Collagen IV	(67)
Pneumococcal antigen	(68)
EBNA	(69)
Entactin Entactin* Phospholipids	(70) (71) (72)

*Mono-specific anti-entactin antibody is included to suggest a control non-cross-reactive antibody to determine if it needs dsDNA as a cross-reactive specificity to gain pathogenic potential.

All these dogmas (i–iv) have been met with controversial hypotheses and experimental results *in vitro* and observations *in vivo*—research that has reduced or completely eliminated their impact *and explained why*. These paradigm shifts have been motivated and introduced by open-minded scientists with the courage to ask critical questions and to search for insightful answers—instead of the premature conclusions: *We know/we knew!*

The new paradigms are the consequences of new and contemplated critical hypotheses that have led to highly relevant experimental and clinical observations. This is further discussed and elucidated below.

Today, we are facing similar conflicts between historical dogmas that contrast the prevailing classification criteria. These conflicts may suggest classification criteria as inferior to strict causal-instigated criteria (3, 45). If taken seriously, these considerations may transform the criteria versions containing many non-coherent measures to fewer concise cause-promoting, interactive, and interdependent criteria linked in a common pathophysiological network—in other words, *diagnostic criteria as complex footsteps of a basic causal etiology* (85).

### 4.1 SLE classification criteria—a relevant historical account

Largely, the procedures we use to define SLE classification criteria follow rules established 50 years ago for the “preliminary SLE classification criteria” (9). In 1971, a strategy for criteria selection principles was chosen without the implementation of the causality principle, and immunological parameters were excluded due to insufficient quality of assay protocols (9).

In 1971, the insight into the immunobiology and pathophysiology of SLE was limited compared with our current (still incomplete) understanding of immunological elements of the syndrome. Despite this fact, we have not changed the early algorithmic approach to generate new classification criteria (9–12, 28). In that context, the algorithms used in 1971/1972 (9, 28) are also used in the most recent classification criteria versions [ (11, 12), see a comparison of SLE classification criteria 1971–2019 in **Table 2**]. This has the inevitable consequence that different published classification protocols are stereotypical and contain reiterated criteria that are *selected but not integrated* in a unified cause-driven inflammatory network [ (31, 86), **Table 2**].

**Table 2:** Comparison* of SLE classification criteria in **four** different classification versions (9–12) from 1971 **to** 2019**.

1971 preliminary SLE Classification criteria.	1982 ACR SLE classification criteria	2012 SLICC SLE classification criteria	2019 EULAR/ACR SLE classification criteria^#^
1. Facial erythema (butterfly rash) 2. Discoid lupus erythematosus **3. Raynaud phenomenon** **4. Alopecia** **5. Photosensitivity** 6. Oral or nasopharyngeal ulceration 7. Arthritis without deformity **8. Lupus erythematosus cells** **9. Chronic false-positive serologic test for syphilis** 10. Profuse proteinuria **11. Cellular casts** 12. Pleuritis or pericarditis 13. Psychosis or convulsions 14. Hemolytic anemia or leukopenia or thrombocytopenia	**1.** Malar rash 2. Discoid rash **3. Photosensitivity** **4.** Oral ulcers 5. Synovitis 6. Serositis 7. Neurologic manifestations 8. Renal manifestations 9. Hematologic manifestations 10. **Immunologic manifestations:** **Anti-DNA/anti-Sm antibodies** Anti-phospholipid antibodies 11. **ANA**	**Clinical criteria:** **1.** Acute cutaneous lupus 2. Chronic cutaneous lupus 3. Oral ulcers: palate **4. Non-scarring alopecia** **5.** Synovitis involving two or more joints or tenderness in two or more joints 6. Serositis 7. Renal disorder 8. Neurologic disorder 9. Hemolytic anemia 10. Leukopenia (<4,000/mm3 at least once) 11. Thrombocytopenia (<100,000/mm3) at least once **Immunological criteria:** 1. **ANA above the laboratory reference range** 2. **Anti-dsDNA above the laboratory reference range** 3. **Anti-Sm** 4. Antiphospholipid antibodies 5. **Low complement** 6. Direct Coombs test	**Obligatory entry criterion antinuclear antibodies** **1. Constitutional fever** **2.** Acute cutaneous lupus 3. Subacute cutaneous OR discoid lupus 4. Oral ulcers **5. Non-scarring alopecia** **6. Joint involvement** **7.** Pleural or pericardial effusion 8. Acute pericarditis 9. Proteinuria >0.5 g/24 h 10. Renal biopsy class II OR V lupus nephritis 11. Renal biopsy class III OR IV lupus nephritis 12. Delirium 13. Seizure 14. Psychosis/delirium 15. Autoimmune hemolysis 16. Leukopenia 17. Thrombocytopenia 18. **Anti-dsDNA antibodies** 19. **Anti-Sm antibodies** 20. **Anti-cardiolipin OR** **anti-ß2GPI OR lupus anticoagulant** 21. **Low C3 OR low C4 low C3 and low C4**

*This table compares the four major SLE classification criteria that appeared from 1971 till 2019. In this table, only criteria without comments or weighted values are given.

**Color code:

*Criteria written in blue are unique for autoimmunity and inflammation parameters and included in the 1982, 2012, and 2019 SLE classification criteria, but not in the 1971 preliminary SLE classification criteria.

*Criteria written in red are shared by some but not all criteria versions.

*Those criteria written in black are shared by all four criteria sets. Criteria may here be designated differently although they express the same or familiar symptoms. For example “Renal manifestations, criterion # 8 in the 1982 ACR criteria, is in the 2012 SLICC criteria designated Renal, criterion # 7, and in the EULAR/ACR criteria denoted Proteinuria >0.5 g/24 h (criterion # 9), Renal biopsy class II OR V lupus nephritis (criterion # 10), and Renal biopsy class III OR IV lupus nephritis (criterion # 11). These versions of criteria contain many of the same individual classification criteria and are differently annotated. These differences reflect increased insight into each criterion and thereby different annotations, and they express the same contextual meaning.

The EULA/ACR SLE classification criteria presented in this table: only criteria are given, for domains, see (12).

### 4.2 SLE classification versus SLE diagnostic criteria—an attempt to define the problem

The process responsible for selecting SLE classification criteria has been described in the original literature (9–12) and in complementary publications [see, e.g (87, 88), with emphasis on the role of Delphi panels (31, 86)]. In the first round, a large number of criteria were in general suggested among selected experts, and in the next round, fewer criteria were elected through democratic votes in Delphi panels (87, 88). The latter sets of criteria were regarded as essential in SLE.

In this process, priority was not given to an authoritative discussion focusing on *what* the criteria were expected to impact or to solve. In the context of the causality principle, the criteria reflect responses (i.e., symptoms or criteria) assumed to be caused by a dominant (SLE-restricted)? stimulus; i.e., they are assumed to be dictated by a dominant etiological stimulus [see the critical discussion in (3, 45)]. Central in this context is the ideal role of coherent and interdependent factors/processes like autoimmunity (17, 33, 89–94), target antigens *in vivo* (5, 17, 34, 35, 65, 67, 77, 95, 96), complement activation and consumption (97–100), and direct effect on target organs [kidneys (5, 101–105), skin (106–109), and cerebrum (110–113)]. *Importantly*, *this ties complexity to causality* (85) and is further contemplated in different contexts below.

Hohmann et al. underscore the importance of expert surveys like the Delphi panel methodology (88). This implies that criteria were selected by democratic processes (criteria with the most votes over a limit are included) and not by prioritized pathogenic and relevant hypotheses. This method “*allows the survey of experts in a high quality and scientific manner. Level V evidence (expert opinion) remains a necessary component in the armamentarium used to determine the answer to a clinical question*” (88). Here, it is not clear whether the Delphi panel-based selection of classification criteria brings an important parameter to the discussion forum: the cause that defines the origin of the symptoms (i.e., criteria). This is another component of the armamentarium, which, however, is not implemented in the critical processes aimed to select the SLE classification criteria. Here, we observe the contour of new alternative algorithms:

- The symptoms (criteria) are caused by an SLE instigating factor.
- The criteria belong to a relatively uniform responsive interdependent pathophysiological network.
- The “indicative criteria” [the circumstantial evidence (114)], as opposed to the guilty cause(s) [see below and (14, 15, 114, 115)], may not be part in the discussion of this interdependent cause-related pathophysiological network. Indicative criteria may, however, promote SLE *imitations* (46, 47) as they *de facto* are implemented in the SLE classification criteria—but not in the group of cause-related hard evidence (meaning diagnostic criteria, see below).
- According to the attribution rules, criteria count whether appearing simultaneously or one by one over undefined time lapses (the accumulative model). This is in clear conflict with Witebsky’s (116) Koch-derived (117, 118) postulates to define disorders as caused by autoimmunity and infections, respectively, and is consequently in conflict with the causality principle!

These aspects are not problematized in the original literature on SLE classification criteria (9–12).

In contrast to the implementation of the causality principle in the criteria selection process, criteria as those used today were in the first round nominated by insight and experience and then elected by democratic processes among the originally nominated criteria (Delphi panels). This has resulted in an inconsistent and complicated discussion of whether SLE, as defined by these criteria, is one genuine disease entity or not.

This problematic discussion involves the following problems (45):

- Whether the classification criteria are coherent and interrelated in a common inflammatory fate destiny was not implemented as a prioritized discussion. This is evident when we study the original literature that focuses on the elaborated SLE classification criteria over the last 50 years (9–12).
- This problematic situation has evolved because the classification criteria promote and embrace a variety of (potentially disparate) syndromes. Some of these criteria may not be linked to a given dominant SLE-specific etiology. These latter criteria may rather promote SLE-imitating disorders.

These contemplations may open for the deduction that cohorts established by recent classification criteria embrace syndromes that simultaneously comprise disparate versions of SLE-like syndromes. These are not secured in clear definitions, hypotheses, or research strategies.

## 5 Controversies in SLE research: three central but unresolved SLE problems that can be decrypted if we consider the combination of the causality principle and relevant historical scientific data

In the following, problems linked to the nature of pathogenic processes that characterize SLE will be discussed. This discussion directly opens for testable hypotheses and for new viewpoints related to concrete explanations and new paradigms. This may be helpful to understand what SLE is: “genuine SLE” separated by concrete facts from the less distinct “SLE-like non-SLE” syndromes.

The enormous amount of scientific literature on SLE (27) is aimed i) to define SLE, ii) to explain the kind of pathogenic category SLE belongs to, and iii) to describe if the symptoms (here: synonymous with criteria) making up this syndrome follow the laws for causality or not.

### 5.1 Definition of SLE

In the emerging concepts of SLE, it is not easy to observe reflections in the relevant literature aimed to critically revise classification criteria and the current definition(s) of the syndrome. A critical revision can open new perspectives for *in-vitro* and *in-vivo* experiments and hypothesis-based clinical observations. These may provide information intended to describe today’s fragmentary understood processes—*processes that cause, modify, or perpetuate SLE*. These processes embrace the regulation of tolerance, the effect of autoimmunity on the generation of symptoms, the mono- and polygenetic bases for SLE or SLE-like disorders, and the impact of infections on the course, outcome, and prevalence of SLE. Promising elements involved in such processes, and possible (semi-)causal therapies, have in recent years been published (2, 8, 16, 17, 41, 64, 110, 119–128).

If the hypothesis that promotes SLE as “a prototype autoimmune syndrome” is correct, then *why are not criteria causally linked to, e.g., anti-chromatin B- and T-cell autoimmunity selected and prioritized over other more “indicative” criteria* (e.g., alopecia, serositis, and arthritis, see point below).

### 5.2 What kind of syndrome category is SLE—the etiology problem

SLE as a “prototype systemic autoimmune syndrome” is an insufficient definition. Autoimmunity as described in the classification criteria (**Table 2**) is by itself a criterion or a symptom that characterizes the syndrome. Autoimmunity is not discussed as a primary causal factor that *instigates* the symptoms or classification criteria—and in the end the syndrome SLE itself. Humoral anti-chromatin autoimmunity is a disease-*modifying* factor in SLE and has its own etiology, but is, according to the classification criteria, not *a sine qua none* in SLE (6, 8, 35, 129, 130).

SLE is linked to several predisposing factors, like monogenic (20–23) and polygenic (24, 25, 131, 132) abnormalities or combinations, sex/gender (133), and environmental factors (134–136). Autoimmunity is in this context not a predisposing factor but a disease-modifying factor. The basic etiology that represents the fundamental predisposition factor(s) may be of genetic origin, but the symptoms are driven by, e.g., autoantibody-mediated inflammation, where immune complexes promote downstream inflammatory networks as central factors in different organs.

In this regard, the criteria promoted by autoantibody-mediated inflammation appear to be caused by the autoantibodies and not directly by the basic genetic predisposition. However, the clinical impact of autoantibodies is in harmony with the causality principle to explain organ inflammation and pathobiological aberrations. They can therefore—with some limitations—be accepted as diagnostic criteria. With these simple words, we have to go upstream in the pathophysiological network to describe the innermost initial (genetical)? factor that promotes and maintains SLE as a chronic relapsing syndrome. In this context, we have to define which symptoms (or criteria) that do belong to the basic inflammatory network *and which do not*. Is the cause for lupus nephritis and arthritis the same or disparate and unlinked? This is a crucial question if we want to develop diagnostic criteria that may define SLE.

### 5.3 Is it possible to identify criteria that reflect the basic etiology(ies) of SLE—implementation of forensic science-related definitions of causal and circumstantial evidence

The causality principle states that every disease measure has a cause that is unique, reiterated, and identifiable. This leads us to identify criteria that appear interrelated and interdependent in a coherent inflammatory network. *Such disease measures are analogs to forensic science definitions of the hierarchy of evidence* (14, 15, 114, 115)*—as is the rationale in a criminal case*. These have different weighted values as “hard” evidence and “circumstantial” evidence (the latter is in the context synonymous with “informative indicators”). Problems related to a hierarchic grading of evidence in a causality context are discussed by Clarke et al. (14) and the central principles of causality in modern science by Bunge (15). Such categorical levels of evidence progress from concrete events (the factual criminal act, or in analogy: the factual pathophysiological etiology, i.e., the initial responsible executer of the pathogenic processes). This terminology is instructive and highly relevant if we try to develop SLE classification and diagnostic criteria. Diagnostic criteria can be regarded as direct consequences of the factual pathophysiological etiology (114, 115, 137).

### 5.4 Hard versus circumstantial evidence—definitions and principles

In a forensic investigation, the “hard” evidence (first-order SLE criteria, see below) points directly to the original indisputable causal factor. Additional indications may inform about the causal factor but will inherit a less central role as circumstantial indicators (or second-order SLE classification criteria). In a wider framework, as in an SLE context, the circumstantial indicators are represented by assumed SLE criteria unlinked from the concrete cause, are less stringent but interpretable though indirectly, and may also indicate alternative explanations [see an extended principal discussion in (138)]. Principally, however, circumstantial indicators should not be excluded from implementation in the holistic research process. They may allow researchers to *assume* that a physical event has occurred as an intended causal act and *may* lead the investigation in the right direction. As in forensic science, medical science intends to explain the character and origin of a vital clinical problem—like understanding the basis for the progressive or remitting nature of SLE. If the criteria emerge unlinked from a basic unified SLE etiology, they serve as circumstantial evidence and may therefore *also* hint at central alternatives: SLE-like imitations (i.e., “SLE-like non-SLE syndromes”). In this situation, some classification criteria may, because they do not reflect the causality principle, result in incorrect iatrogenic^2^ classification processes and ultimately in inconsistent SLE cohorts.

If we read the relevant literature, it is clear that separate SLE-related clinical and basic scientific disciplines, and their promoting scientists, do not communicate sufficiently and open-mindedly with each other through explorative and informative cross-talks and collaborations. This affects the most central elements of the SLE syndrome. Examples to be discussed here are amenable to hypotheses and prioritized as follows: i) deficient connection between the causality principle and SLE classification criteria; ii) definition of “the anti-dsDNA antibody,” i.e., one single specificity without further distinctions; and iii) pathogenic impact of an autoantibody (exemplified by anti-dsDNA antibodies)—hypotheses versus evidence relevant to understand the molecular basis for their pathogenicity. All necessary information has been available in the literature for decades that can be implemented to solve the three problems and is discussed in detail below. This information has been largely ignored and not considered in current research hypotheses or scientific analyses.

## 6 What kind of pathophysiological parameters reflect causality—and are their impact ignored in the legitimation of classification criteria?

In the following, some central examples will be discussed in terms of the schism between clinical viewpoints and relevant evidence-based historical and basic science information. If these two elements could be combined through discussions and collaborations across scientific borders, this would have the potential to generate new paradigms and new innovative hypothesis-driven research efforts.

### 6.1 SLE classification criteria: are they hypothesis-driven, and why is the causality principle not implemented—theoretical arguments for grading and categorizing criteria

From what we know—and do not know—about the syndrome, SLE will inevitably precipitate the central question: Is SLE, as we prefer to think today, a definable syndrome with a unified etiology—in other words, a “disease entity” (31)? Today, two possible hypotheses exist: SLE as a “genuine” syndrome driven by one dominant cause or a vaguely defined polyphenotypic group that *also* implements “SLE-like non-SLE disorders.” The latter may be difficult to sort out as “non-SLE” syndromes. Ultimately, is this a discussion of *classification versus diagnostic criteria* and in a wider sense a discussion of *phenomenology versus causality* [discussed recently in (139)]?

Identification of downstream SLE classification criteria without sufficient consideration of upstream causal factors (the causality principle) is problematic. Classification criteria-defined SLE is not an unambiguous diagnosis, as we still have not been able to develop a firm definition of the syndrome beyond the recurrently used idiom “*SLE is an enigmatic and a prototype systemic autoimmune syndrome.*” From a scientific reality, many phenotypically different variant disorders may have been classified as SLE (including SLE imitators).

#### 6.1.1 First-order SLE criteria reflect a basic causal etiology

From the perspective of SLE as a polyphenotypic syndrome, some of these variants may theoretically be regarded as “genuine SLE” on one side and as imitators of SLE (46, 47) on the other. The SLE-imitating variants may be interpreted as disparate “*SLE-like non-SLE disorders*.” This contemplation may help to sort out a condition that represents “genuine SLE.” Genuine SLE fulfills a definition that ideally may implement an autoimmune, anti-chromatin antibody-driven pathogenic etiology with a corresponding consequent and interdependent downstream inflammatory network and disparate, although coherent, organ manifestations (5). This model implies the basic hypothesis that first-order criteria (as hard evidence, see above) directly reflect a basic etiology linked to a gene-based abnormality that may predispose to autoimmunity [e.g., monogenic SLE (20–23)].

#### 6.1.2 Second-order SLE criteria serve as circumstantial indicators

Criteria like alopecia, serositis, and arthritis do not belong to the same cause-related criteria network basically promoted by anti-dsDNA antibodies. They may therefore, in forensic scientific terms (114, 115), be accepted as second-order criteria (as circumstantial indicators) not directly reflecting the basic etiology of SLE. This grading principle has not been contemplated in the relevant original literature (9–12). If the complete sets of SLE classification criteria belong to a unified network of inflammatory parameters were not even tentatively discussed in these reports (9–12).

#### 6.1.3 Classification criteria and SLE cohorts—what characterize them

Each individual SLE cohort that appears based on the attribution rules, as defined in the classification criteria literature, is per definition polyphenotypic irrespective of whether it represents.

- “a one disease entity,” which then per definition may have one dominant etiological factor—i.e., according to the “*one etiology-disparate phenotypes-one disease entity*” paradigm—or.
- a classification process that promotes cohorts that have different phenotypes and etiologies—the *polyetiological paradigm including “genuine SLE” and “SLE-like non-SLE syndromes.”*.

In any case, the attribution rules, as defined in contemporary classification criteria publications, generate cohorts with SLE patients presenting different clinical phenotypes, like with or without anti-dsDNA antibodies and with or without nephritis, cerebral lupus, and so on. This problem is real both from theoretical considerations (as a derivation of the attribution rules) and also when looking at the composition of published cohorts [discussed in (3, 33, 45)].

In one study, Isenberg et al. enrolled 988 SLE patients in a flare study (140). According to the results, they were able to categorize patients into eight dominant phenotypic groups. They state: “*Case histories were carefully reviewed and assigned into 1 of 8 clinical groups: musculoskeletal and/or skin disease only, joint and/or skin and renal disease, mainly serositis, mainly renal, mainly gastrointestinal, mainly central nervous system, joints and/or skin plus serositis, and other, which included predominantly hematologic and/or constitutional or other combinations.*” These results, in the present author’s opinion, may indicate eight different SLE versions that may be understood as an argument against SLE as a “one disease entity.” A naive question in this context: The eight categorized groups including many SLE patients point at disparate clinical phenotypes; do these groups of patients emerge by different etiologies or by one dominant etiology? If one dominant etiology, the enrolled patients could theoretically belong to “*the one etiology-disparate phenotypes-one disease entity*” paradigm. If they arise from different etiologies, the eight groups may *also* comprise “SLE-like non-SLE syndromes”.

If “the” SLE syndrome (in singular) is real with respect to a definable delimitation toward potential imitators, this may clearly define SLE as a “one disease entity.” This will precipitate the simple, somewhat naive but complicated question: What is SLE and what is not SLE? This puts a clear critical focus on the following questions:

- What is the scientific explanation that allows us to accept various random combinations of criteria, like any 4 out of 11 archetypical ACR classification criteria (10), to define SLE as “a one disease entity”^3^; the same idea for classification principles are recommended in the other classification criteria versions.
- Why are the classification criteria meant just for classification, while they in fact contribute as diagnostic criteria: All patients enrolled into a classified SLE cohort are per definition and attribution rules in practice claimed to suffer from SLE. Consequently, classification criteria circumnavigate diagnostic criteria and take over their function and impact.

SLE is still an enigmatic and complex syndrome. The causality principle states that every effect—here defined as a disease measure or a criterion—has a cause (15, 141, 142). This should be implemented in the future development of causally related (diagnostic) criteria that characterize the syndrome SLE.

### 6.2 Is “the anti-dsDNA antibody” a clinically relevant and unambiguous term—and are they all always clinically important?

*Wolfgang Goethe once stated: “The hardest thing to see is what is in front of your eyes”* ^4^.

This phrase is highly relevant for us in the following context. The mainstream rheumatology literature claims anti-dsDNA antibodies to be a unique diagnostic marker and a central pathophysiological factor in SLE [see, e.g (5, 8, 10–12, 35, 143–145)]. It is a remarkable observation that systematic scientific studies on DNA structure, biology, and immunity have demonstrated that each of several unique DNA structures is immunogenic and is able to induce pertinent structure-specific antibodies. This statement is based on data collected over the last seven! decades [reviewed in (34)]. The referred classical studies collectively demonstrate that “the anti-dsDNA antibodies” do not embody one single specificity that possesses diagnostic and pathogenic impacts. DNA constitutes a diversity of distinct, functional, and unique DNA structures (33, 34, 51, 52, 54, 146–151), which all are individually immunogenic. This information is more or less ignored or disregarded in the contemporary mainstream literature on diagnostic rheumatology and clinical immunopathology, although some few exceptions from this viewpoint exist (see below).

Different DNA structures and their relevant biological functions have been described [see below and (152–154)]. Basically, their individual and unique roles are to direct, support, execute, and regulate DNA repair, replication, and transcription of genes. Notably, the structures have a striking yet largely disregarded relevance in an autoimmune context: Each structure has, aside from their basic DNA-associated functions, a unique ability to induce highly specific and segregated antibodies, *both in experimental and autoimmune contexts* [for details, see (34) and a concise and condensed information below].

Thus, from a series of published studies over more than seven decades, it is clear that established, concise insights into DNA structures and functions tell a quite different story than that about the sole existence of “the anti-dsDNA antibody” (34). This history contrasts the general clinical view about anti-dsDNA antibodies operating as a single antibody specificity (9–12, 28–30). In fact, Rosalind Franklin was the first to describe the unique forms of DNA beyond its pure helical structure in 1953: the A- and B-DNA (155).

Another simplification of the history of anti-dsDNA antibodies is that they are claimed specific for SLE [thoroughly and objectively discussed in (6, 8, 34)]. This is evidently wrong! Anti-dsDNA antibodies are not unique for SLE [see **Figure 1A**, discussed in (6)] but occur regularly in infections (35, 83, 156–163) and malignancies (84, 164–170) and sporadically in other disorders [see, e.g (157, 171)].

Data published over the last decades imply that anti-dsDNA antibodies specifically recognize concise DNA structures like elongated and highly bent mammalian B-DNA, Z-DNA, ssDNA, cruciform DNA, and infectious viral and bacterial DNA, with high precision (see **Figures 1B, C**, discussed in (34)]. The diagnostic and/or pathophysiological impact of these subspecificities has neither been determined nor studied systematically. These antibodies are detected in specific assays as exemplified in **Figure 1C**.

#### 6.2.1 Central DNA structures and their immunogenic and pathogenic potential—insight into problems that are largely ignored in SLE research

When we shall try to understand the pathogenic potential of anti-DNA antibodies, we need to settle the premises for this discussion. The following elements are central: specificity, nature of the immunogenic structures (and their counterparts, the *in-situ* target), immunogenicity, and complement-activating potential. This is not a clear-cut and simple paradigm. The specificity of anti-DNA antibodies is the history of ignored DNA structure–function relationship in clinical immunology. We have to put these antibodies into new connections: the diagnostic and pathogenic framework and perspective.

In the following, a condensed summary of analyses will be communicated, with a focus on structures, functions, and immunogenicity of intrinsic elements of mammalian DNA [see **Figure 1B** (152–154)] with theoretical challenges to modify our comprehension of anti-dsDNA antibodies as a diagnostic and pathogenic factor.

*B-DNA* is the most disseminated DNA structure in the human genome. The composition (172, 173) and structure of the B form DNA as a right-handed double helix (155, 174, 175) reflect in many ways the basic dsDNA in its relaxed low energy and resting conformation. B-DNA is immunogenic in both experimental and spontaneous clinical situations like in SLE, cancers, and infections (6). B-DNA presents two different structural versions, each linked to unique functions: elongated DNA and bent B-DNA. *Elongated B-DNA* (synonym for linker DNA) is a stretched linear form of B-DNA. Its name defines its context, a link between core nucleosomes. This creates the iconic electron microscopy picture of “beads on a string” as demonstrated by Olins and Olins in 1974 (176). The histone octamer and histone H1 bind to the linker DNA and contribute to the unmasking of genes and to chromatin compaction (177). Like H1, the histone octamers (2x(H2A,H2B,H3,H4)) slide along B-DNA and form *bent B-DNA*, the part of DNA wrapped around the histone octamer (178–181). This formation facilitates the effects of regulatory proteins like high-mobility group proteins to bend DNA into various degrees of flexible conformations (182–184). Studies on kinetoplast DNA [a network of circular DNA (185)] have demonstrated that certain sequences cause DNA to be highly bent and that other sequences bend in response to the binding of proteins (186). The bent form of B-DNA is formed as a recurrent structure in chromatin.

The *ssDNA structure* appears in two different contexts: i) as *intended functional ssDNA or not-intended* denatured ssDNA in analytical contexts and ii) stabilized opened transcriptionally active ssDNA (187, 188). Single-stranded DNA-binding proteins (SSBP in **Figure 1B**) hold the ssDNA intact and are exposed during the course of its function: DNA transcription, recombination, and repair (189), and serve as template for opposite strand DNA synthesis (190).

*Z-DNA* is structurally and functionally integrated in the human genome (191, 199–201) and is involved in various human diseases [see (192, 193) and references therein]. Z-DNA differs from the B-DNA structure: it is a left-handed, high-energy supercoiled double helix. Physiologically, Z-DNA forms *in vivo* during transcription (194) that depends on the interaction of mobile polymerases and other regulatory proteins (195, 196). Z-DNA participates directly in the regulation of the rate of transcription.

*Cruciform DNA* is different from B- and Z-DNA. Its formation requires that inverted sequences (palindromes) present in one strand are repeated on the other strand in the opposite direction. This promotes the formation of hairpin and cruciform DNA structures. The cruciform structures are like Z-DNA, higher-energy DNA structures that are important for regulating biological processes (197, 198).

**Figure 2:**
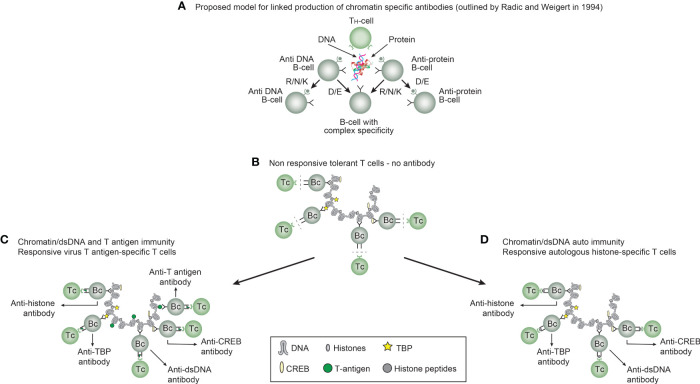
Models for the production of anti-DNA and anti-chromatin antibodies. In order to understand the results of experimental and empirical studies aimed to describe the origin of anti-dsDNA antibodies, we need to settle a semantic distinction: Anti-dsDNA antibodies may be the result of immune responses to DNA–protein complexes in two different contexts: *immunity versus autoimmunity* **(A–D)**. In general, antibodies to dsDNA generated *in vivo* are most probably a result of both categories of immunity. There are many reasons to argue for the validity of these models to generate anti-DNA antibodies. The arguments were basically presented as a theoretical model for the future by Radic and Weigert in 1994 [presented in **(A)**]. In this model, aspects of affinity maturation are demonstrated as the B-cell Ig variable regions are undergoing mutations to basic or acidic residues [panel **(A)** is redrawn from a figure in reference (41) and is provided courtesy of Dr. Marko Radic, University of Tennessee Health Science Center]. Derived from this theoretical model, functional evidence-based models by Marion et al., Gilkeson et al., and Rekvig et al. are demonstrated (42–44, respectively). In the absence of responsive T cells, a model for tolerance is presented **(B)** and implies no T-cell help for DNA/chromatin-specific B cells. The distinction between immunity and autoimmunity is demonstrated in **(C, D)**, respectively. The principal difference relies on the specificity of the T cells. In immunity, the T cells are specific for, and engaged by, non-self-derived DNA-binding proteins [like polyomavirus T antigen in panel **(C)**], while in autoimmunity, the T cells are engaged by autologous, chromatin-derived proteins like histones **(D)**. The basic model promoted by Radic and Weigert predicts a molecular and cellular prototype model also for the linked production of antibodies to DNA, histones, and other chromatin-associated proteins. The repertoire of chromatin-specific autoantibodies is from theoretical considerations the same for the models presented in **(C, D)** (see text for details). This instructive and informative figure is copied from reference (34).

*Immunogenicity of DNA structures*: Unique DNA structures have a clear potential to induce highly segregated and structure-specific antibodies and can serve as targets for such antibodies *in vivo* (**Figure 1B**). Available information concludes that the individual DNA structures in isolated DNA or in chromatin all may be accessed and consequently can interact with B cells (afferent immunogenic stimulus), thus rendering them immunogenic. The model in **Figure 2A** was proposed by Radic and Weigert in 1992 and predicted the combined production of a spectrum of chromatin-specific antibodies (41, 199–201). This model was later verified, as principally shown in **Figures 2B–D**, by Marion et al., Rekvig et al., and Pisetsky et al. [extensively reviewed in (6)]. In **Figures 2C, D**, this cognate model is validated for polyomavirus T antigen-specific T helper cells (**Figure 2C**) and for true autoimmune histone-specific T helper T cells (**Figure 2D**).

Derived from these models, the induced anti-dsDNA antibodies may consequently recognize and access the same chromatin ligands as those recognized by the B-cell antigen receptors in germinal centers. The latter efferent effect explains its pathogenic effects. This is true both for the experimentally induced antibodies (as in **Figure 2C**) and spontaneously produced autoantibodies [**Figure 2D** (6, 8),]. Immunity to cruciform DNA has been induced experimentally, while autoimmunity to this structure has still not been reported. Despite this basic insight, the clinical (diagnostic and pathogenic) impact of these structure-specific anti-DNA antibodies has not been sufficiently investigated.

These specificities must in a clinical context be handled individually and not as a single unit (as “the anti-dsDNA antibody”) because they are specific only to the discrete structures that induce them [reviewed and discussed in (34, 54, 199)]. It is here important to stress that we still have not determined the clinical (diagnostic and pathogenic) impact of these individual antibody specificities.

#### 6.2.2 Specific anti-DNA antibodies and selective assay conditions

We do not critically consider which specificities we test for by the different clinically relevant analytical assay principles. Structure-specific anti-dsDNA antibodies can be analyzed by specifically designed assay principles [see examples in **Figure 1C** and in reference (34)]. This is important to consider in future studies as we now need to describe these anti-dsDNA antibody subspecificities in diagnostic, classification criteria and pathogenic contexts. As some of these specific antibodies are easier to induce experimentally than others due to differences related to how they are controlled (tolerated) by the immune system (51, 53, 54, 200, 201), they may therefore differ in diagnostic impact and pathogenic potential. This may also partly be due to the variable density of the unique structures along the DNA helix as they appear in the chromatin structure (34).

### 6.3 Pathogenic impact of an autoantibody: how does this comply with the causality principle

The anti-dsDNA antibodies play important but controversial and inconsistent roles in immunology (6, 8, 33, 35, 41, 42, 92, 129, 202), molecular biology (54, 121, 203–207), and rheumatology, as well as in infections and malignancies (33, 79, 82–84, 163, 169, 208–211).

An international consensus states that autoimmunity in clinical contexts complies with two central aspects: diagnostic and/or pathogenic factors. Both are in current scientific literature based on paradigms that on one side rely on facts and on implementation on the causality principle and on the other side on simplifications and irrational dogmas, as SLE classification criteria are the gold standard—SLE diagnostic criteria are rejected from clinical practice [discussed in (4, 45)].

For example, in Goodpasture syndrome, the direct clinical effect of autoantibodies binding collagen 4 is nephritis and alveolitis (212, 213). This cause–effect relationship is documented in basic, mechanistic studies (213).

**Figure 3:**
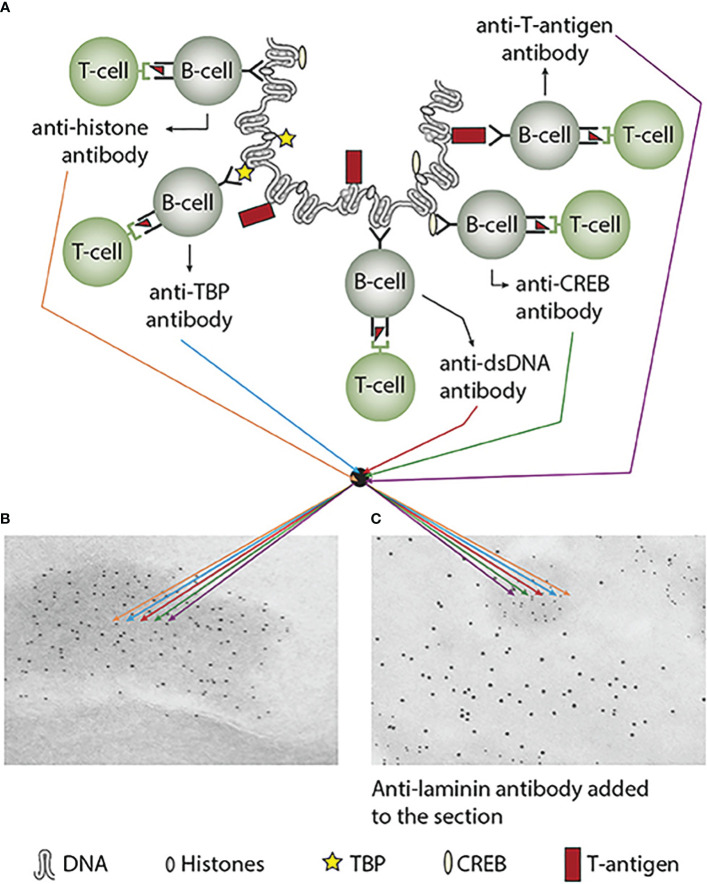
Experimental induction of anti-dsDNA antibodies and other chromatin autoantibodies by *in-vivo* expression of a single viral dsDNA-binding protein. In **(A)**, injection of normal mice with plasmids encoding wild-type polyomavirus DNA-binding T antigen in the context of eukaryotic promoters predictively induced the production of antibodies to T antigen and significant production of antibodies to mammalian dsDNA, histones, and to certain transcription factors like TATA-binding protein (TBP) and cAMP-responsive element-binding protein (CREB). All autologous chromatin-derived ligands physically linked to T antigen can therefore be rendered immunogenic to autoimmune B cells that present peptides derived from T antigen in the context of MHC class II molecules. Therefore, concerted production of autoantibodies specific for chromatin antigens, including dsDNA and histones, is not dependent on an SLE-related background but may appear also in quite healthy individuals. In **(B)**, the group of chromatin autoantibodies notably including anti-dsDNA antibodies targets exposed chromatin in the kidneys. As demonstrated by immune electron microscopy, it is evident that the autoantibodies target the electron-dense structure (EDS), convincingly demonstrated to constitute chromatin fragments [(36–38), see left immune electron microscopy picture (antibody binding is observed as 5-nm gold particle-labeled autoantibodies in **(B)**]. These autoantibodies did not bind clean GBM structures (seen as clean membranes surrounding the EDS) or the mesangial matrix (not shown). However, anti-laminin antibodies added to the sections *in vitro* bound clean GBM (observed as 10-nm gold particle-labeled antibodies), and they did not co-localize with *in-vivo*-bound anti-chromatin antibodies [5-nm gold particles in **(C)**]. These data argue for the fact that anti-dsDNA/anti-chromatin antibodies bind chromatin fragments *in vivo*, and they did *not* bind inherent, regular membrane components. This latter observation is an argument against the cross-reactive model for lupus nephritis. This figure was first published in reference (5).

In a larger autoimmune perspective, two factors must access each other to fulfill the pathogenicity of an autoimmune response. These two crucial factors are i) the autoimmune antibody and ii) a cognate and accessible target antigen. This clearly identifies a problem in autoimmunity: the presence of, e.g., an anti-dsDNA antibody, does not per se indicate a pathogenic effect of the antibody. **Figure 3** presents a principle model that implements how anti-chromatin antibodies are produced (as explained in **Figures 2A–D** and detailed in **Figure 3A**). In **Figure 3B**, the group of chromatin autoantibodies targets exposed chromatin in GBM. As demonstrated by immune electron microscopy, it is evident that the autoantibodies target an electron-dense structure (EDS), demonstrated to constitute chromatin fragments [ (36–38), by immune electron microscopy in **Figure 3B** **).** Antibodies bound *in vivo* are here stained by 5-nm gold particles. These autoantibodies did not bind clean GBM structures surrounding EDS or the mesangial matrix. However, anti-laminin antibodies added to the sections *in vitro* bound clean GBM (seen as 10-nm gold particle-labeled antibodies), and they did not co-localize with *in-vivo*-bound anti-chromatin antibodies (5-nm gold particles, **Figure 3C**). These data argue for the fact that anti-dsDNA/anti-chromatin antibodies bind GBM-associated chromatin fragments in the context of lupus nephritis, and they do *not* bind inherent, regular membrane components.

The cognate antigen *in vivo* in this model is the chromatin complex. This complex is located in the nucleus, ergo hidden for the antibody. This implies that two processes must be fulfilled: i) externalization of chromatin and ii) reduced clearance of extracellular chromatin. The latter is achieved in situations where, e.g., renal DNase 1 expression is reduced (36–38). In this situation, undigested, large chromatin fragments are exposed in, e.g., the mesangial matrix and in the glomerulus basement membranes. In other words, the two factors can access each other, form immune complexes, and promote, in this exemplified context, lupus nephritis. This conforms to the causality principle. In the absence of the chromatin fragments, the anti-dsDNA antibodies must be regarded as a pathophysiological epiphenomenon—but may still serve as a diagnostic criterion. This scenery explains why not all anti-dsDNA antibodies exert a pathophysiological process.

How does this picture comply with cross-reacting autoantibodies? Also, in this context, autoantibodies toward mammalian B-DNA are a great example, as anti-mammalian B (ds)DNA is promiscuously cross-reactive (6, 34). The pathogenic impact of cross-reactive anti-dsDNA antibodies is noticed, discussed, and assumed but poorly defined with one possible exception: cross-reactivity with alpha-actinin (55, 78). In many studies describing the cross-reactivity of anti-dsDNA antibodies, it is *assumed* that the interaction of the antibodies with the cross-reactive antigens may explain their pathogenic impact. This is at best an insufficient assumption if this cross-reactive process is not examined by descriptive and experimental analyses. Cross-reactivity by itself does not inform about which of the cross-reactive antigens are targeted *in vivo*.

The pathogenic potential of an autoantibody relies on two central prerequisites: i) complement-activating Ig classes and IgG subclasses and ii) the density of the targeted epitopes. The latter needs to be implemented, as for IgG antibodies, the steric density of IgG Fc-regions is essential to activate complement (98).

#### 6.3.1 The causality principle and its influence on targeted, causal treatment

“The causality principle” implies that the inflammatory process is explained by which antigens are targeted by the antibodies *in vivo*. Antibodies that recognize exposed, extracellular chromatin fragments promote a type III immune-mediated disease (214) that can be treated by anionic compounds like heparin and heparinoids (39, 125, 128). This is demonstrated in lupus-prone mice treated with heparin. In treated mice, the production of anti-dsDNA antibodies was reduced and delayed, as was the nephritic process in these mice. The reason for this effect is that heparin opens the compacted chromatin fragments and makes them sensitive to proteases and nucleases (39). This leads to a reduced antigenic load *in vivo*. Altogether, the heparin-related data provide an alternative basis for semi-specific and semi-causal treatment of lupus nephritis (39, 124, 125, 128, 215, 216).

If the antibody binds a cross-reactive membrane structure like Alpha-actinin, laminin or entactin [see references (55, 59, 61, 70, 73, 74, 76, 78, 217, 218)], this will promote a type II immune-mediated disorder (214). This interaction is principally different from the mechanism exerted by complexes of anti-dsDNA antibodies and chromatin fragments. The therapeutic heparin model described here is unfunctional in the cross-reactive model, and till now, no causal treatment is known for this model. Importantly, therefore, the mechanistic impact of the two conflicting models, type II and type III immune-mediated tissue inflammation, has not been comparatively investigated in concise and systematic prospective studies. Such studies are important to conduct because each model may have unique and individual gateways to causal therapy modalities.

In conclusion, the causality principle—the cause and its consequent effect—is important to implement to understand the basis for pathogenic processes and also to prepare hypotheses aimed to describe causal therapeutic modalities.

There is still a need to perform collaborative projects between groups that promote each of the two models to clarify which model indeed is correct or if there exist overlaps between them. This is a central problem that needs to be solved as the two models may be treated differently.

## 7 Concluding remarks

This author’s critical approach is based on studies and evolving arguments over the decades. These arguments are discussed in recent articles (2–5, 8, 122, 140) and are basically relevant to implement in order to criticize the mode of classification criteria selection. Till now, no firm pathogenic or genetic arguments validate if each of the criteria in fact tells us if and how they adhere to SLE (45, 139), and everything in this text converges into the term causality.

*Interconnected and interactive pathophysiological processes* may evolve as a consequence of one cause and may form the epitome of an understandable syndrome. Provided that the etiology is described and understood, the ensuing symptoms/criteria are ideally predictable, recognizable, reiterated, and explicable in the patient [one cause always results in the same effect, and the effects may act as new causes with new downstream effects (or symptoms/criteria)]. This means that the cause promotes predictable downstream criteria that can be utilized as finger-pointing diagnostic criteria. Furthermore, in a reverse situation, these criteria may hint at a cause, but not always.

In an SLE context, the epitome “equal causes promote equal effects”^5^ is for theoretical and practical reasons important, while equal effects do not necessarily equal cause(s). This rule proclaims *predictability and identity if the cause is initial or reiterated.* That is, we can anticipate the effects of a given cause; the opposite, to anticipate a cause by given effects, is problematic since an effect may originate from disparate causes.

*The search for an etiology (or a cause)* responsible for *de-facto* observed SLE criteria is compatible with the term *reversibility*: That is to say, we can reverse the *process* both intellectually and informationally. This can also be reformulated by an example as follows: Proteinuria in an SLE patient hints at the increased production of anti-dsDNA and anti-chromatin antibodies and a simultaneous loss of renal DNase 1. In this sense, the pathophysiological effects may (reversely) point to immune complexes of chromatin and anti-dsDNA antibodies that serve as the cause of nephritis in this SLE patient. This is logical and understandable for the SLE category dominated by anti-dsDNA antibodies and the exposure of chromatin fragments. *For other criteria, we have to validate first their link to SLE through biological processes, and then to make collections of criteria growing out of a single root (etiology). Thus, for individual criteria, we need first to identify theoretical and testable causes for their appearance before they can be accepted as first-order, hard evidence criteria.*

Indeed, we have still a long way to go to transform the enigmatic SLE syndrome into (a) rational, understandable disorder(s). After all these years, SLE is still an enigmatic syndrome, and we still do not know the clinical impact of the many autoantibodies observed in SLE (219).

## Data availability statement

The original contributions presented in the study are included in the article/supplementary material. Further inquiries can be directed to the corresponding author.

## Author contributions

OPR: Conceptualization, Data curation, Formal analysis, Funding acquisition, Investigation, Methodology, Project administration, Resources, Writing – original draft, Writing – review & editing.

## Footnotes

2 Iatrogenic means “a complication induced unintentionally by a physician or surgeon or by medical treatment or diagnostic procedures” (Merriam-Webster).

3 In fact, a random combination of 4 out of 11 SLE classification criteria [ACR SLE classification criteria (10)] will theoretically result in 330 clinical SLE phenotypes (45). Whether all these phenotypes adhere to “a one disease entity” is far from being established.

4 Translated from German: “Was ist das Schwerste von allem? Was dir das Leichteste dünket. Mit den Augen zu sehen, was vor den Augen dir liegt”.

5 For details, see The Principle of Causality, in *Principia Cybernetica.*
